# Metabolic Adaptation during Cardiac Exercise Rehabilitation in Patients after a First Myocardial Infarction

**DOI:** 10.1021/acs.jproteome.5c00997

**Published:** 2026-02-03

**Authors:** Eleonora Bossi, Marta Nobile, Federico Paoletti, Lorenzo Ticini, Simone Serrao, Alessia Giglio, Gianfranco Parati, Antonio Zaza, Lia Crotti, Gabriella Malfatto, Giuseppe Paglia

**Affiliations:** † Department of Medicine and Surgery, 60233 University of Milano-Bicocca, Monza 20900, Italy; ‡ 9354 IRCCS, Istituto Auxologico Italiano, Department of Cardiology, Cardiac Rehabilitation Unit, S. Luca Hospital, Piazzale Brescia 20, Milan 20149, Italy; § Department of Biotechnology & Bioscience, 9305 University of Milano-Bicocca, Milano 20126, Italy

**Keywords:** metabolomics, lipidomics, dried blood spots, acute myocardial infarction, cardiac rehabilitation, physical training

## Abstract

Cardiac rehabilitation (CR) is highly beneficial in postmyocardial infarction (MI) patients; however, its metabolic impact remains underexplored. This study investigated metabolic and lipidomic adaptations to an intensive CR program in 25 nondiabetic male patients (<75 years) following a first uncomplicated ST-elevation MI (STEMI). CR involved 24 ± 3 sessions, with baseline and final clinical assessments, and, in a subgroup of 17 patients, longitudinal dried blood spots (DBS) were collected, and metabolomics/lipidomics analysis was also performed. CR significantly improved clinical outcomes, including the 6 min walk test, B-type natriuretic peptide (BNP), left ventricular ejection fraction (LVEF%), C-reactive protein (CRP), and homocysteine levels. Metabolomic analysis showed sustained metabolic adaptations, notably increased *N*-acetyl-l-tyrosine (NAT), suggesting a mitohormesis response to exercise-induced mitochondrial stress. The third training session exhibited the highest metabolic adaptation, primarily in energy metabolism pathways like the TCA cycle, indicating enhanced oxidative energy generation and improved exercise performance. The lipidome displayed an acute response to the first training, with upregulation of phosphatidylserines (PS). Predicted increased activity of phosphatidylserine synthase-1 (PSS1), enzymes vital for PS synthesis, underscores PS’s protective role in myocardial damage and its contribution to muscle activity. These findings highlight CR’s beneficial metabolic adaptations, potentially via mitohormesis, and suggest possible mechanistic targets and candidate biomarkers requiring investigation in future controlled intervention studies.

## Graphical abstract

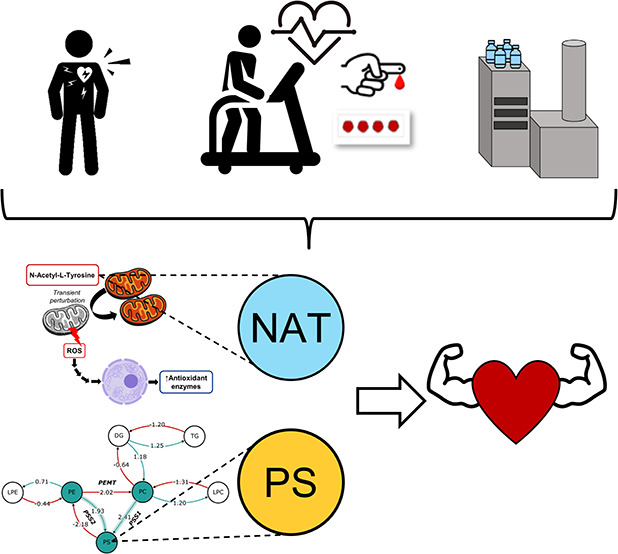

## Introduction

Ischemic heart disease remains the leading cause of death worldwide.^1^ The beneficial effects of cardiac rehabilitation (CR) through physical exercise on the progression of the disease in postacute myocardial infarction patients are well-known.^2–4^ CR has numerous beneficial cardiovascular, metabolic, psychological, and functional effects, overall improving both cardiovascular outcomes and overall patient well-being.^4,5^ Among the metabolic effects, CR improves lipid profile^6^ and glucose metabolism;^7^ it also reduces inflammatory status.^8^ However, a more systemic view of its effects on the body’s metabolic processes is still missing.

Mass spectrometry (MS)-based metabolomics and lipidomics can provide a systemic view of the body’s metabolic response to exogenous or endogenous stimuli by analyzing the complete set of small molecules present in a biological system. Several studies demonstrated that physical activity can cause metabolic changes in both healthy and clinical populations, affecting energy metabolism, lipids, and amino acid pathways.^9–11^ Indeed, we also previously demonstrated that the MS-based metabolomics and lipidomics can capture a more systemic metabolic adaptation to physical exercise under both hypoxic and hyperoxic conditions.^12–14^ Moreover, we have implemented procedures for coupling blood microsampling with metabolomics to facilitate longitudinal studies where multiple sampling is required.^15–20^

While metabolomics has been used in several studies to describe the metabolic signatures of cardiovascular events,^21–23^ to the best of our knowledge, only one study to date has employed metabolomics and lipidomics to explore the effects of myocardial infarction followed by CR in six subjects, with an assessment conducted one year postischemic event.^24^ However, this long-term effect might not capture the metabolic adaptations occurring during and immediately after CR.

This study was designed as a hypothesis-generating pilot investigation of metabolic and lipidomic responses to CR.

In this study, we followed the metabolic response during a 6-week period of intensive CR in selected male patients who had suffered a first myocardial infarction (MI): first, the metabolomic and lipidomic state was analyzed before and immediately after the whole CR period; moreover, the metabolomic and lipidomic state was also studied before and after a single bout of exercise at three different periods during CR, by obtaining multiple collections of dried blood spot (DBS) samples. Blood microsampling techniques have been successfully used to profile metabolic and lipidomic responses to acute exercise. Cendali et al. detected changes in glycolysis, TCA cycle, and lipids in response to a running exercise bout using microneedle-based DBS.^25^ Likewise, DBS metabolomics captured a broad range of exercise-induced metabolic changes, including glycolysis, fatty acid metabolism, and the TCA cycle. Similar alterations were observed in plasma as well, supporting DBS as a viable alternative to profile exercise-induced adaptations.^26^

## Materials and Methods

### Materials

All extraction solutions and UHPLC solvents were LC–MS grade—LiChrosolv—and were purchased from Merck KGaA (Darmstadt, Germany): water, MeOH, ISO, ACN. Medronic acid and ammonium acetate were purchased from Sigma-Aldrich/Merck (Darmstadt, Germany), as well as EquiSPLASH LIPIDOMIX Mass Spec Standard (Avanti Polar Lipids, Alabaster, AL, USA).

### Study Population and Rehabilitation Protocol

Among patients referred to our Cardiac Rehabilitation Unit from July 2023 to July 2024 after a first noncomplicated myocardial infarction (MI), we included 25 consecutive subjects with the following characteristics: male gender, age 25–70 years, and absence of any of the following exclusion criteria: peripheral vascular disease, diabetes mellitus, hemodynamic instability, heart failure (NYHA class II–III), moderate/severe chronic obstructive pulmonary disease, inability or impossibility to understand or accept the informed consent. The limited and homogeneous study population reflects the hypothesis-generating nature of the study and was selected to minimize biological and clinical confounding. Table 1 summarizes the relevant baseline characteristics of all the patients.

**Table 1:** Baseline Characteristics of the Study Population

Clinical data	
*N*/males	25/25
Age (years)	57.2 ± 13.6
STEMI/NSTEMI	23/2
Site (anterior or anterolateral vs inferior)	16/9
Complete revascularization	21/4
Left ventricular ejection fraction (LVEF) (%)	48.6 ± 7.9
Wall Motion Score Index (WMSI)	1.7 ± 0.3
Global Longitudinal Strain (GLS) (%)	–13.1 ± 3.3
6 min walking test at the beginning of CR (6MWT) (meters)	559 ± 73
Hematochemistry	
BNP, pg/dl	171 ± 128
CRP, mg/L	0.8 ± 0.9
Homocysteine, mmol/L	15.1 ± 4.2
Folate, ng/mL	5.7 ± 4.5
Ferritin, ng/mL	316,6 ± 149.3
Glycated hemoglobin (HbA1c), %	5.6 ± 0.5
LDL-cholesterol, mg/dl	57.9 ± 20.0
Lipoproteine a (LP_a_), nmol/L	109.7 ± 80.6

The CR program was organized by the team of cardiologists and physiotherapists and performed on an outpatient’ basis, following our internal protocol based on European and Italian guidelines:^4,27^

The protocol had a duration of 5 to 7 weeks (mean 6.1 ± 0.8 weeks) and consisted of three to five sessions per week (average 3.5 ± 0.7 sessions/week), each lasting 90 min.

Overall, patients were exposed to 22 ± 2 sessions (range: 20–24 sessions).

Each session included 15 min of warm-up and stretching exercises, 45 min of aerobic interval training on the cycle ergometer or treadmill. Finally, 30 min of medium-intensity calisthenics.

The interval training was modulated based on the results of the 6 min walking test (6MWT) on the day of entry. The 6MWT was performed in accordance with accepted best clinical practice,^28,29^ with patients walking for 6 min along a 25 m hallway.

The variables monitored throughout the test were as follows: peripheral O_2_ saturation and heart rate (HR); arterial blood pressure was measured at the beginning and at the end of the test. The target HR for exercise training was calculated as suggested by Calegari et al.,^29^ i.e., HR-6MWT was used as the training rate: we adjusted the exercise intensity, i.e., the training load, in order to reach and maintain the HR-6MWT during the whole training session. In the course of each session, subjects were monitored with ECG and were under cardiological supervision.

6MWT, blood tests, and echocardiography were performed at the beginning and at the end of the CR program. In detail, echocardiography was executed using color Doppler and Tissue Doppler Imaging (TDI).^30^ The echo parameters considered were the left ventricular ejection fraction (LVEF), the global longitudinal strain (GLS), and the wall motion score index (WMSI). The hematochemical parameters considered were C-Reactive Protein (CRP), homocysteine, folate, ferritin, B-type natriuretic peptide, glycated hemoglobin (HbA1c), Low-Density Lipoprotein cholesterol (LDL-c), and Lipoprotein a (Lp­(a)).

### Metabolomic and Lipidomics Sample Collection and Preparation

Blood samples for metabolomic and lipidomics analysis were collected during the 6 weeks of the CR protocol at 6 time points using a DBS Whatman 903 Protein Saver Card (Cytiva, Global, Little Chalfont, UK). Complete data sets for all time points are available from 17 of the 25 enrolled patients. Incomplete data sets derived primarily from logistical constraints inherent in the clinical rehabilitation setting, including missed sampling time points, limited patient availability for blood collection, and incomplete adherence to the full rehabilitation protocol. Sampling included measurements taken 15 min before and 15 min after: (1) the first training session (week 1), (2) the first training session of the second week, and (3) the final training session. DBS samples were collected, dried for 2 h, and then stored at −80 °C (Figure 1). DBS sampling with nonvolumetric devices may be affected by hematocrit-related effects, which may alter extraction recovery due to differences in blood viscosity and spreading. However, no hematocrit correction was applied, as validation studies without hematocrit correction have described acceptable analytical performance over physiological ranges for different analytes.^31,32^

**Figure 1:**
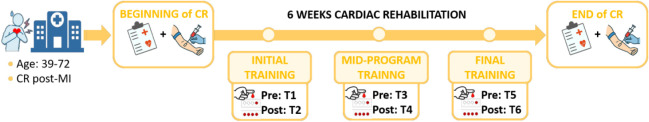
Experimental design. At the beginning and end of the CR protocol, all subjects underwent a medical examination and a series of routine clinical tests. The CR protocol lasted 6 weeks. DBS samples were collected at 6 time points pre- and post-training in three different weeks during the CR protocol: at the beginning of rehabilitation, halfway through the rehabilitation, and at the end of the rehabilitation.

DBS samples were processed as previously described.^15^ In brief, immediately after removal from the −80 °C storage, two 3 mm-diameter spots were punched from each DBS sample and transferred to fresh 1.5 mL Eppendorf SafeLock tubes, which were incubated on ice for 30 min. A first extraction was performed by adding 400 μL of MeOH 100%. Samples were then stirred for 20 min at 4 °C and 450 rpm in a ThermoMixer Compact (Eppendorf, Hamburg, Germany) and then centrifuged for 15 min at 4 °C and 21,000*g*. Supernatants were collected and divided into two equal aliquot volumes to be used for polar metabolites and lipids assays, respectively. A second extraction was performed by adding 80 μL of water to the sample and repeating the stirring and centrifugation steps. The supernatant recovered from this second extraction was added to the aliquot for polar metabolite analysis. All samples were filtered with 3 K cutoff filters (Millipore Amicon) Ultra 0.5 mL, Merck KGaA, Darmstadt, Germany). Filtration was performed with 3 cycles of centrifugation at 14,000*g* at 25 °C for 15 min. For lipidomics analysis, 6 μL of deuterated EquiSPLASH LIPIDOMIX Mass Spec Standard was added to each sample as an internal standard. Samples were then freeze-dried for 2 h at RT using the HetoVac VR-I (A. De Mori, Milan, Italy) and reconstituted with 80 μL of ACN/H2O (50/50 v/v) for polar metabolites and 80 μL of isopropanol (ISO) for lipids. Quality control (QC) samples for both polar metabolites and lipids were prepared by pooling 2 μL aliquots of the extracts obtained after the individual extraction of each dried blood spot sample.

### UHPLC-MS Analysis

The analysis was performed using a UHPLC-MS platform comprising an Agilent 1290 II liquid chromatography system coupled to a quadrupole time-of-flight mass spectrometer (Agilent 6546 LC/Q-TOF—Agilent Technologies, Palo Alto, CA, USA).^15^ Chromatographic separation for polar metabolites was achieved using an InfinityLab Poroshell 120 HILIC-Z (2.1 × 150 mm, 2.7 μm) column (Agilent Technologies, Palo Alto, CA, USA) equipped with a UHPLC InfinityLab Poroshell 120 HILIC (2.1 × 5 mm, 2.7 μm) guard column. Mobile phase A consisted of 20 mM ammonium acetate and 5 μM medronic acid in water. Mobile phase B was made of pure ACN. The following solvent gradient was used for sample elution: 0 min 90% B, 1 min 90% B, 8 min 78% B, 12 min 60% B, 15 min 10% B, 18 min 10% B, and 23 min 90% B, at a flow rate of 0.4 mL/min. Chromatographic separation for lipids was performed using a CSH ACQUITY Premier C18 (2.1 × 100 mm, 1.7 μm) column (Waters, Milford, MA, USA). Mobile phase A consisted of 10 mM ammonium acetate in ACN/H_2_O (60/40 v/v) and 0.1% acetic acid. Mobile phase B was made of an ISO/phase A mixture (90/10 v/v). Samples were analyzed at a flow rate of 0.25 mL/min with the following elution gradient: 0 min 99% A, 1 min 99% A, 1.10 min 60% A, 5 min 20% A, 11 min 20% A, 12 min 1% A, 18 min 1% A, 18.10 min 60% A, and 20 min 99% A. The analysis was performed in both positive (2 μL injection volume) and negative (5 μL injection volume) ionization modes. For metabolomics, the resolution was set to 40,000 fwhm with a full scan range of 40–1200 *m*/*z*, while for lipidomics it was set to 50,000 fwhm and operated in a full scan range of *m*/*z* 100–1350. QCs were used to monitor the performance of the analysis and were injected every five samples. At the end of the analysis, five injections of QC samples were used to collect MS/MS spectra in data-dependent analysis (DDA) using an iterative approach.

### Data Analysis

Data Acquisition (Agilent Technologies, Santa Clara, CA, USA) was used to run the Agilent 1290 II liquid chromatograph and the Agilent 6546 LC/Q-TOF mass spectrometer. MassHunter Profinder (Agilent Technologies, Santa Clara, CA, USA) was used for feature annotation. Five consecutive injections of QC samples in DDA mode were used to acquire MS/MS data that were employed along with online databases such as HMDB^33^ and METLIN^34^ to build the in-house library for polar metabolites and lipids based on accurate mass, MS/MS fragments, isotopic pattern, and retention time. Samples analyzed in full scan mode were matched with our in-house library using mass formula, isotope pattern, and retention time, and integrated with MassHunter Personal Compound Database and Library (PCDL) Manager Software (Agilent Technologies, Santa Clara, CA, USA). Univariate and multivariate statistical analyses were performed using MetaboAnalyst 6.0 ^35^ and GraphPad Prism 9.5 (GraphPad Software, Boston, MA, USA, www.graphpad.com), after data were normalized to the sum of the signals and data transformation by log_10_ transformation. Quantitative enrichment analysis for polar metabolites was performed using The Small Molecule Pathway Database (SMPDB),^36^ after normalization to the sum of the signals, data transformation by log_10_ and autoscaling. For lipidomics analysis, the area of each feature was first normalized to the area of the corresponding lipid class in the internal standard, followed by the log_10_ transformation. The fatty acid (FA) class was normalized using the lysophosphatidylcholine (LPC) class from EquiSPLASH, chosen as a surrogate due to similar retention times and physicochemical properties, providing effective normalization in the absence of a dedicated deuterated FA standard. This RT-matched surrogate approach aligns with lipidomics practices that prioritize retention time-matched internal standards when class-specific deuterated analogs are unavailable.^37^

Given the hypothesis-generating and exploratory nature of this study, no multiple testing correction was applied. Therefore, all reported *p-values* should be considered nominal. Lipid enrichment analysis was performed with LION/web.^38^ Moreover, lipid pathway analysis was performed with BioPAN^39^ to investigate systematic changes at different lipid levels and predict a link to gene activity. The BioPAN workflow involves the calculation of the Z-score based on the mean and standard deviation of the experiment, assuming that the data for the lipid subclasses are normally distributed. The Z-score estimates the statistical significance of a change in a certain metabolic reaction between the control and treatment conditions. A global pathway Z-score is determined by combining the Z-scores for all reactions. A reaction or pathway is automatically classified as significantly changed (*p* < 0.05) if Z > 1.645.^39^ If a change is significant, reactions are classified as either activated or suppressed depending on their sign.

## Results and Discussion

### Cardiac Rehabilitation Outcomes

No complications were observed during the CR period. Upon discharge, 23 out of 25 patients had achieved optimal medical treatment, i.e., high doses of statins, ACE inhibitors, beta-blockers, and antidiabetic drugs. Baseline blood levels of folate, ferritin, glycated hemoglobin, LDL-c, and high-sensitivity troponin were already within the normal range and were not changed by CR. Also, no effect of physical exercise on LP­(a) levels was observed, consistent with existing literature.^40^ The parameters that were significantly changed by CR are summarized in Table 2. The impact of the CR program is demonstrated by the improvement in 6MWT, echocardiographic, and hematochemical parameters.

**Table 2:** Effects of 6 Weeks of Cardiac Rehabilitation on Clinical and Hematochemical Data^a^

A—Exercise tolerance	
6MWT (meters)^b^	
Pre CR	Post CR
559 ± 73	614 ± 67

B—Echocardiography					
LVEF (%)^b^		WMSI^b^		GLS (%)^b^	
Pre CR	Post CR	Pre CR	Post CR	Pre CR	Post CR
48.6 ± 7.9	51.2 ± 6.8	1.7 ± 0.3	1.4 ± 0.3	-13.1 ± 3.3	-15.4 ± 3.1

C—Hematochemistry					
BNP (ng/mL)^b^		CRP (mU/mL)^b^		Homocysteine (mU/mL)^b^	
Pre CR	Post CR	Pre CR	Post CR	Pre CR	Post CR
171 ± 128	115 ± 87	0.8 ± 0.9	0.2 ± 0.4	15.1 ± 4.2	13.3 ± 3.2

a Data are presented as mean ± 1 standard deviation for both pre- and post-CR.

b *p*-value <0.05. Only parameters significantly changed by CR are shown.

### Overall Impact of Cardiac Rehabilitation on the Metabolome/Lipidome Profile

Our investigation into the metabolic changes began by assessing the overall impact of the CR program, “integrated” over the six-week period. This was achieved by comparing pretraining DBS samples, collected at admission and before the last training session, respectively. As depicted in the volcano plot of Figure 2A, the CR program was associated with an increase in several metabolites, including specific acylcarnitines and phosphatidylethanolamines, with a log_2_ fold change (FC) ranging between ±0.5. Of particular interest was the marked increase in *N*-acetyl-l-tyrosine (NAT) by the end of the CR protocol. Analysis at the intermediate time points, detailed in the boxplots of Figure 2B, revealed that a significant rise in NAT concentration occurred already after the first training session and was sustained through the following ones (e.g., between the midprogram and final trainings) with only minor (nonsignificant) dips. This is of relevance because NAT has been identified as a key endogenous factor in mitohormesis, the adaptive cellular response to mild stress triggered by mitochondrial release of low levels of reactive oxygen species (ROS).^41^ Physical activity is a recognized mitochondrial stressor, leading to ROS production and downstream adaptive signaling cascades.^42^ While the precise mechanism of mitochondrial stress-induced ROS release in mitohormesis is still being elucidated, NAT has been identified as a key intrinsic activator of this pathway across species (mice and *Drosophila* larvae).^43^ Coherently, NAT pretreatment significantly improved stress tolerance in these models.^43^ Our finding of sustained NAT’s upregulation throughout the CR protocol strongly aligns with these reports and suggests that mitohormesis may represent a significant beneficial outcome of CR. Additional support for this hypothesis comes from the upregulation of allantoin and pyroglutamic acid during the first training session (Figure 3A), as both are associated with oxidative stress,^44–46^ the mitohormesis trigger. Further evidence indicates that exercise-induced mitohormesis is also relevant to human physiology.^42^ Collectively, these results suggest that in the post-MI setting, CR may trigger an adaptive response in oxidative metabolism, potentially contributing to energy homeostasis and its adaptation to physical exercise, a benefit unappreciated thus far.

**Figure 2:**
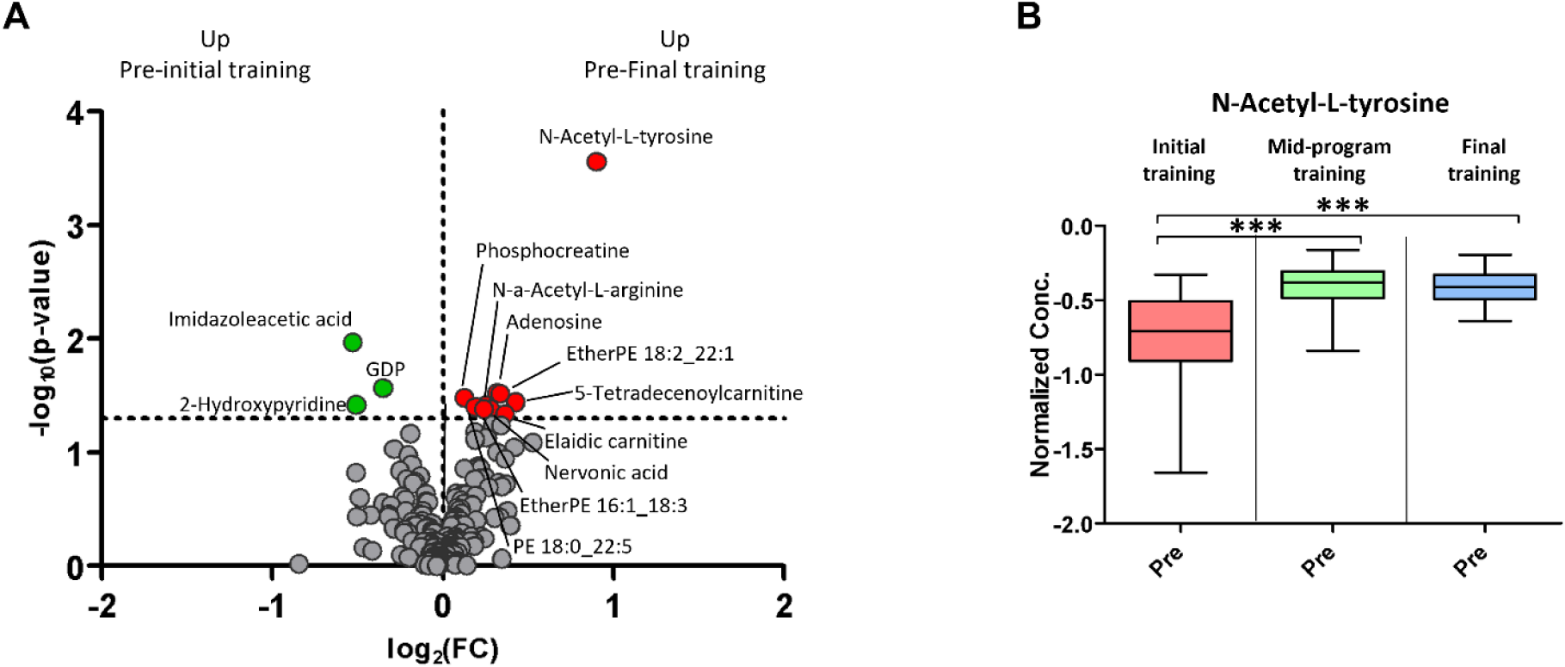
Metabolic changes due to the CR program. A) Volcano plot: preinitial training (beginning of rehabilitation) vs prefinal training (end of rehabilitation) (*p-value* threshold 0.05, FC 1). B) Boxplots: significant changes of *N*-acetyl-l-tyrosine (preinitial training vs premid-program training vs prefinal training), *p*-value threshold 0.05.

**Figure 3:**
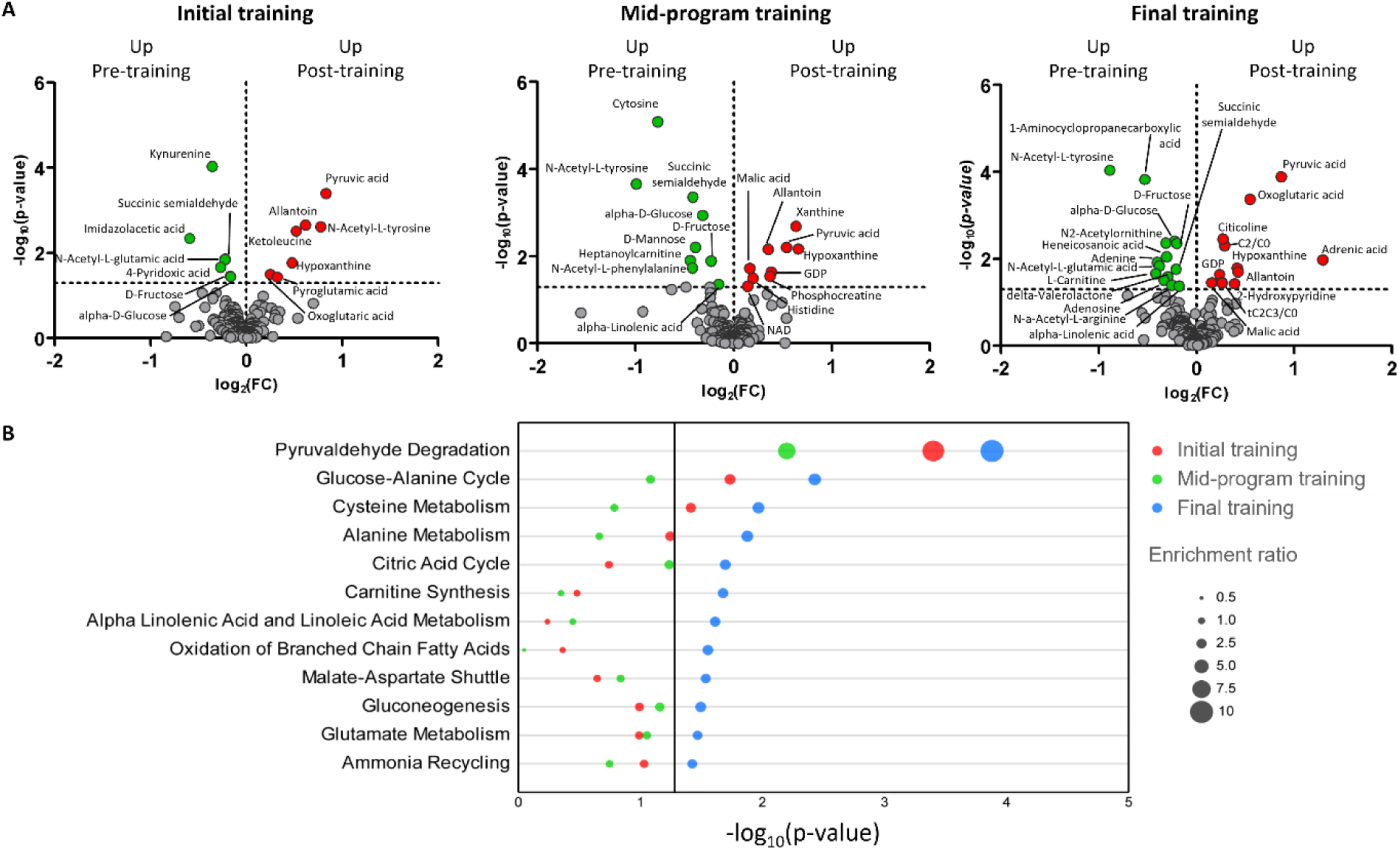
Polar metabolites analysis. A) Volcano plots of initial training, midprogram training, and final training (*p*-value threshold 0.05, FC 1). −log_10_(*p*-value) is on the *Y*-axis and log_2_(FC) is on the *X*-axis. Green dots represent statistically significant metabolites downregulated post-training. Red dots represent statistically significant metabolites upregulated post-training. B) Overlaid quantitative enrichment analysis obtained considering the polar metabolome during initial training (red), midprogram training (green), and final training (blue). Each training implies the pre- vs post-training comparison. Each dot shows how much a single pathway is enriched in the considered comparison. The more enriched the pathway, the bigger the dot, according to the enrichment ratio, which is proportional to the number of metabolites seen in that pathway. Pathway names are shown on the *Y*-axis, while −log_10_(*p*-value) is on the *X*-axis. Only significant pathways (in at least one training) are shown.

### Metabolic Response to Individual Training Sessions

To investigate the impact of single-session rehabilitation training on patients’ metabolism, we performed univariate and multivariate statistical analyses on metabolomics and lipidomics, comparing pre- vs post-training samples at each CR session.

Volcano plots presented in Figure 3 clearly show that the polar metabolome captures a higher metabolic adaptation during the final training session, compared to earlier ones (Figure 3A). Indeed, functional analysis performed by quantitative enrichment (Figure 3B) shows that the final training was the most effective in enriching most pathways, largely related to energy metabolism.

To illustrate the adaptations induced by CR, Figure 4 displays boxplots and FC trends for significantly affected metabolites. Notably, the concentrations of pyruvic acid, hypoxanthine, and allantoin significantly increased with each training session. Statistically significant changes also included an increase in oxoglutaric acid during the initial and final sessions, an increase in malic acid during the midprogram and final sessions, and a decrease in l-carnitine during the final session. Also, the increments of the ratios tC2C3/C0 (tC2C3 = sum of acetylcarnitine (C2) and propionylcarnitine (C3); C0 = l-carnitine) and C2/C0 achieved significance at the final session.

**Figure 4:**
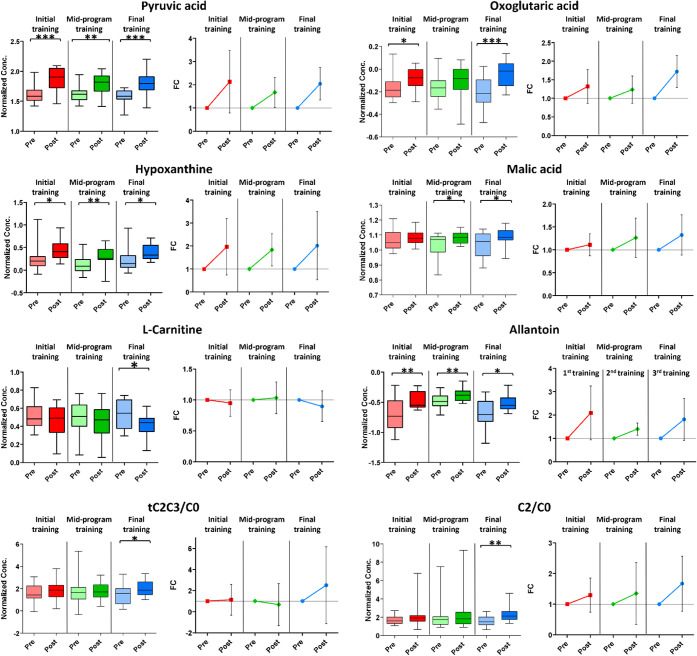
Polar metabolome analysis. Boxplots and FC trends highlight significant changes of polar metabolites (*p*-value threshold 0.05). Initial training in red, midprogram training in green, and final training in blue.

Overall, the polar metabolome highlighted that the most significant changes can be detected after the final training session, thus suggesting a “memory” effect progressively increasing the metabolic response to exercise throughout the CR program.

As shown in Figure 3, such a response mostly concerns key metabolites of energy metabolism; indeed, pyruvic acid, oxoglutaric acid, and malic acid are intermediates of TCA cycle, all linked to oxidative energy production.^12,13,47^

The glucose-alanine cycle encompasses glucose oxidation to pyruvate and further catabolism to alanine (via the glutamate-pyruvate transaminase (GPT)-catalyzed reaction), a pathway fueling the TCA cycle by providing oxoglutaric acid from glutamate. Enhancement of the glucose–alanine cycle is a further element in the adaptive effect of CR training.

It is known that catabolic conditions (fasting or physical exercise) increase the oxidation of fatty acids in mitochondria. Fatty acids enter the cytosol from plasma, are converted into CoA- thioesters, and subsequently are transferred into the mitochondria via the palmitoyl-CoA carnitine transferase II shuttle.^48,49^ The import of acyl-CoA uses l-carnitine (C0), thus resulting in decreased blood levels of free carnitine. Moreover, the increased flux of oxidation in cells accumulates acetyl-CoA, which is then released as acetylcarnitine into blood.^50,51^ Our results are consistent with previous results, where the circulating plasma levels of C0 and the sum of acylcarnitines responded in an almost mirror-like anticorrelation to catabolic challenges.^52^

The concomitant decrease in circulating free carnitine and free fatty acids together with the increase in acylcarnitines is consistent with the improved capacity for mitochondrial fatty acid oxidation and metabolic flexibility. Similar acylcarnitine patterns have been reported in endurance-trained individuals, where enhanced matching between fatty acid flux and β-oxidation demand reflects greater mitochondrial efficiency and training status.^53^ In this context, the present findings suggest that cardiac rehabilitation may induce metabolic adaptations that partially resemble endurance-type responses despite the lower absolute workload and the clinical nature of the population. Importantly, these changes align with the mitohormesis framework supported by a sustained increase in *N*-acetyl-l-tyrosine and the acute elevation of oxidative stress-related metabolites such as allantoin,^43,54,55^ suggesting that repeated moderate mitochondrial stress during rehabilitation promotes adaptive remodeling of oxidative metabolism rather than pathological stress.

Hypoxanthine is part of purine metabolism, which is known to increase with exercise as a consequence of enhanced ATP turnover. Morville et al. reported a stronger association between hypoxanthine levels and resistance training compared to endurance training in healthy individuals, suggesting a link with higher exercise-induced stress and rapid ATP utilization. However, increases in hypoxanthine have also been consistently observed with endurance training, particularly rising early postexercise, indicating a potential dependence on training intensity.^56^ Consistent with this, Contrepois et al. observed great, transient increases in hypoxanthine immediately after acute exercise, highlighting the temporal sensitivity of purine metabolism to exercise-induced stress.^57^ According to recent studies, the blood concentrations of purine derivatives, especially hypoxanthine, are indicators of the training status in elite athletes. Indeed, purine metabolism reflects the response to physical exercise and skeletal muscle adaptations better than cardiorespiratory and other biochemical indicators.^58^ Consistent with previous studies, we found that the level of hypoxanthine was increased after each of the training sessions of the CR protocol. Given the impaired oxidative capacity characteristic of post-MI subjects, moderate-intensity CR may elicit metabolic responses comparable to those observed with higher-intensity workloads in healthy individuals. Together, these findings support the relevance of hypoxanthine as a sensitive marker of exercise-induced metabolic stress in clinical rehabilitation settings and suggest its potential utility as a candidate biomarker for CR monitoring.

Physical exercise is also associated with oxidative stress, and it has been shown that, besides mitochondria, cytosolic and membrane xanthine oxidoreductase and NADPH oxidase contribute to ROS production during exercise.^59^ We found increased blood levels of allantoin after each training session. This metabolite is also involved in the purine metabolism since it is the final product of uric acid oxidation by ROS, and it has been proposed as a potential biomarker of oxidative stress.^54^ Moreover, its concentration does not depend on that of uric acid, thus increasing its specificity as a reporter of oxidative stress.^55^ In addition, in a cohort of subjects undergoing high-intensity exercise, allantoin concentrations were found to be higher in muscle and twice as high in plasma after training.^55^ Overall, these results may suggest allantoin plasma levels are another potential candidate biomarker for CR monitoring.

Altogether, these results likely reflect increased oxidative energy generation by beta-oxidation, maintenance of TCA cycle efficiency, and oxidative phosphorylation at the end of the rehabilitation program.

This effect was mainly evident at the end of the program, suggesting either a progressive increase in performance during CR or greater exercise intensity tolerance by the end of the protocol, leading to greater metabolic perturbation. Overall, the polar metabolome captures a sustained positive metabolic adaptation, increasing cellular energy provision, a crucial factor in improving exercise performance (Table 2) and might translate into functional benefits and metabolic health through increased mitochondrial oxidative capacity.

We then evaluated the impact of each session of rehabilitation training on the lipidome of patients enrolled in this study. Our analysis revealed that CR training significantly impacts the lipidome of enrolled patients, with the most pronounced changes occurring after the first training session (Figure 5A, Volcano plots). Specifically, we observed an upregulation of several phosphatidylserine (PS) lipid species in the blood following the initial training (Figure 5A). This finding was further supported by lipid ontology enrichment analysis using LION/web, which confirmed the overall upregulation of the PS lipid class after the first session (Figure 5C). Beyond PS, phosphatidylinositols (PI) also showed upregulation after the first training. Conversely, free fatty acids (FA) and phosphatidylcholines (PC) were downregulated following the first training session (Figure 5C). Functional analysis using BioPAN Lipidmaps, comparing post- and prefirst-training data, predicts an increased activity of two key enzymes: Phosphatidylserine Synthase 1 (PSS1) and Phosphatidylserine Synthase 2 (PSS2) (Figure 5B). The heightened activity of these enzymes suggests an enhanced conversion of phosphatidylethanolamines (PE) and phosphatidylcholines (PC) into phosphatidylserines (PS).

**Figure 5:**
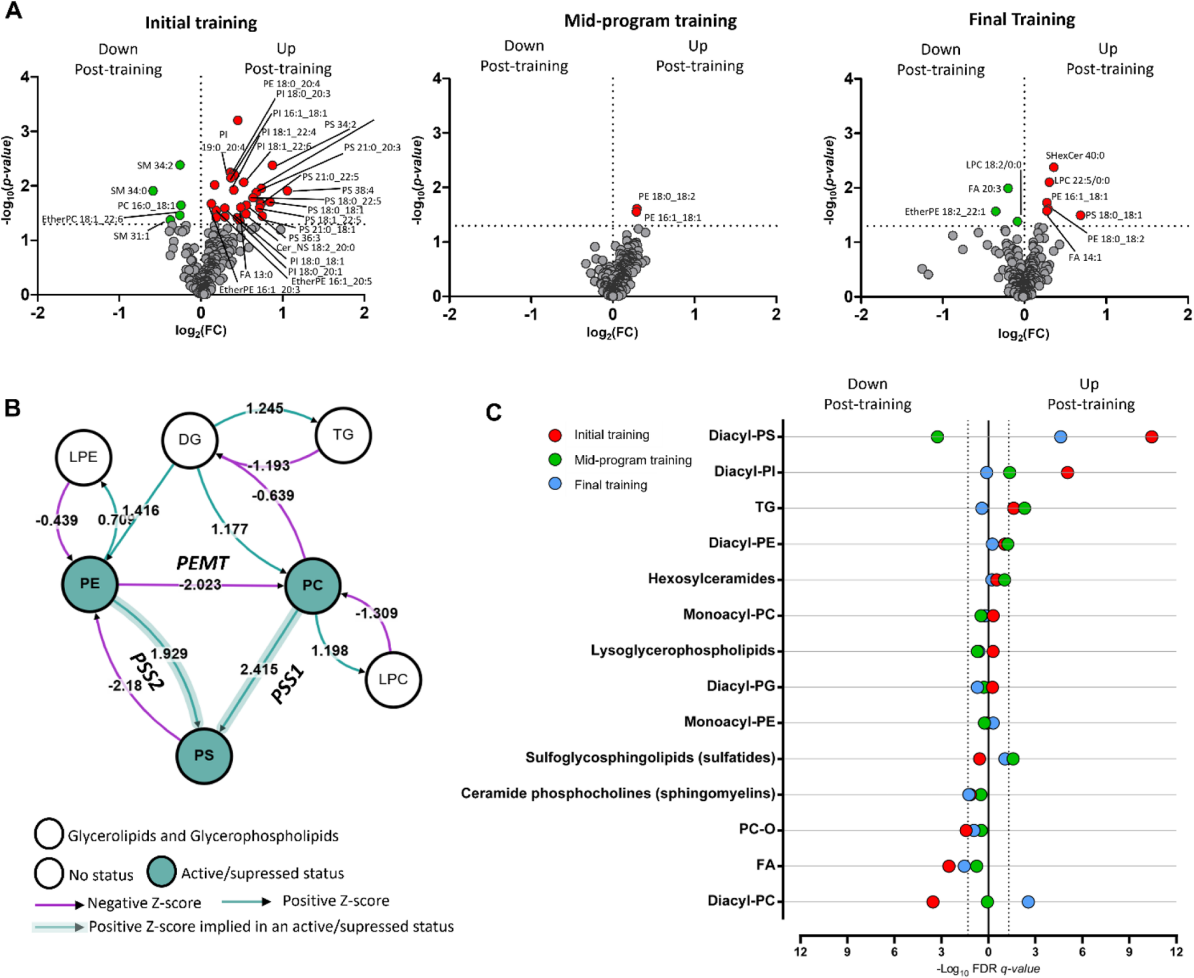
Lipids analysis. A) Volcano plots of initial training, midprogram training, and final training (*p*-value threshold 0.05, FC 1). B) Functional analysis performed using BioPAN LipidMaps on post- vs preinitial training (T2 vs T1), with T2 chosen as the condition of interest. Reactions converting PE and PC into PS were predicted to be more active just after the initial training with *z-*scores of 1.929 and 2.415, respectively, predicting upregulation of PSS1 and PSS2. C) Lipid ontology enrichment analysis using LION/web, considering initial training (red), midprogram training (green), and final training (blue). Each training implies pre- vs post-training comparison. Lipid class names are shown on the *Y*-axis, while −log_10_FDR­(*q*-value) is on the *X*-axis. Dots to the right of value 0 on the *X*-axis represent lipid classes that are upregulated post-training. Dots to the left of value 0 on the *X*-axis represent lipid classes that are downregulated post-training.

Recent studies highlighted the importance of glycerophospholipid metabolism in myocardial infarction (MI).^60,61^ Cardiac PS levels and PSS1 expression were decreased following MI in mice, and overexpression of PSS1 restored PS levels and prevented cardiomyocyte apoptosis, thus suggesting that maintaining adequate PSS1 expression could be crucial in mitigating myocardial damage after MI.^60^ Accordingly, oral supplementation of PS in a mouse model increased the cardiomyocyte survival by 50% in acute myocardial ischemia-reperfusion and reduced the infarct size by 30% in chronic MI. The authors suggested that the main responsible mechanism might be the upregulation of protein kinase C epsilon (PKC-ε), the main player of cardioprotection during preconditioning.^61^ Therefore, PS enrichment and prediction of PSS1 upregulation by CR disclose its potential in attenuating the global burden of MI, perhaps as a preconditioning intervention. To summarize, previous works have shown that MI may have a short-term lipidomic effect, mostly consisting of downregulation of glycerophospholipid metabolism and, in particular, PS levels. According to the present findings, CR may reverse the situation by upregulating PSS1, thus restoring PS levels from the PC pools. PS may also support skeletal muscle activity during physical exercise.^62–65^ In addition, PS supplementation has been shown to modulate cortisol responses following moderate-intensity exercise.^66^ Since cortisol concentrations were not assessed in the present study, potential links between PS and cortisol cannot be evaluated. This represents a limitation of the current study. Including cortisol measurements in future investigations would give insight into the relationship between PS supplementation, cortisol modulation, and exercise-induced physiological responses. Thus, it could be interesting to evaluate cortisol levels and explore the related PS response in further studies.

Although an acute PS response to the initial exercise stimulus was observed, it did not persist throughout the CR protocol. Acute responses are commonly observed in exercise-induced metabolic and lipidomic adaptations as a consequence of the exposure to a novel physiological stress, followed by attenuation as training adaptations occur. Large-scale multiomics studies have shown that several metabolites and lipid species, including membrane phospholipids, exhibit marked transient increases immediately after exercise that reduce during recovery and with repeated exercise exposure.^57^ In this context, the early PS elevation may represent a stress-induced signal, initiating the adaptation process. As training progresses and metabolic efficiency improves, training may elicit reduced energetic and cellular stress, decreasing the need for acute PS mobilization. Therefore, a PS response early in CR may still contribute meaningfully to adaptation without requiring persistence throughout the protocol.

## Limitations

This study has several limitations. The small sample size (*n* = 17) with complete data and the absence of a control group limits statistical power and causal inference. Additionally, the study population was restricted to nondiabetic male patients under 75 years old with a first uncomplicated MI at a single rehabilitation center, which may limit generalizability. Hematocrit was not corrected in DBS sampling, which may affect absolute concentrations, detectability, and recovery of specific metabolites, although previous validation studies suggest the impact is acceptable. The relatively short follow-up period also prevents the assessment of the longer-term persistence of the observed metabolic changes. It remains unclear whether metabolic normalization would continue, plateau, or decrease after CR completion. Finally, cortisol concentrations were not measured, so the potential link between PS, cortisol response, and exercise-induced metabolic adaptations cannot be evaluated. Considering the absence of multiple testing corrections, the present findings should be interpreted cautiously and require confirmation in larger cohorts. Future studies addressing these limitations are required to confirm and extend the present findings.

## Conclusions

The current study analyzed the changes in the metabolic balance induced by a brief period of cardiac rehabilitation focused on intensive physical training after the first uncomplicated myocardial infarction.

As expected, rehabilitation improved overall patients’ condition, as suggested by increased exercise tolerance, better left ventricular function, lower BNP levels, and reduced biomarkers of the general inflammatory response, such as CRP and homocysteine.^8^ We found that CR was associated with changes in the polar metabolome compatible with the cumulative improvement of mitochondrial oxidative capacity, likely of long-term significance. On the other hand, the lipidome describes a short-term upregulation of PS synthesis, which has been suggested to play a role in reducing infarct size and preserving cardiac function. The nature of the observed changes and the finding that NAT progressively increased over the training period point to a role of mitohormesis in orchestrating the observed response to CR. These results identify potential mechanistic targets and candidate biomarkers that warrant investigation in future controlled intervention studies. Despite the relatively small sample size, the findings generate novel mechanistic hypotheses on the CR benefit and indications for supplementation and monitoring. The findings encourage further studies of larger populations and additional measurements of cardiac performance; specifically designed experiments are required to validate the mechanistic significance of the correlative evidence provided by the study.

## Supplementary Material



## Data Availability

Raw data supporting the results are available at the following link through an FTP/SFTP client: ftp://MSV000098996@massive-ftp.ucsd.edu.

## Notes

Data on all pairwise comparisons (preinitial vs prefinal training) and corresponding *p*-values for both polar metabolites and lipids (XLSX)

## Abbreviations

CAN: acetonitrile
BNP: B-type natriuretic peptide
C0: l-carnitine
C2: acetylcarnitine
C3: propionylcarnitine
CR: cardiac rehabilitation
CRP: C-reactive protein
DBS: dried blood spots
DDA: data-dependent analysis
FA: fatty acid
FC: fold change
GLS: global longitudinal strain
HbA1c: glycated hemoglobin
HR: heart rate
ISO: isopropanol
LDL-c: low-density lipoprotein cholesterol
Lp­(a): lipoprotein A
LPC: lysophosphatidylcholine
LVEF: left ventricular ejection fraction
MeOH: methanol
MI: myocardial infarction
NAT: *N*-acetyl-l-tyrosine
PC: phosphatidylcholine
PE: phosphatidylethanolamine
PI: phosphatidylinositol
PKC-ε: protein kinase C-epsilon
PS: phosphatidylserine
PSS1: phosphatidylserine synthase 1
PSS2: phosphatidylserine synthase 2
QC: quality control
ROS: reactive oxygen species
STEMI: ST-elevation MI
TCA: tricarboxylic acid cycle
TDI: tissue doppler imaging
UHPLC-MS: ultrahigh performance liquid chromatography–mass spectrometry
WMSI: wall motion score index
6MWT: 6 min walking test
